# Predicting Surgery Targets in Temporal Lobe Epilepsy through Structural Connectome Based Simulations

**DOI:** 10.1371/journal.pcbi.1004642

**Published:** 2015-12-10

**Authors:** Frances Hutchings, Cheol E. Han, Simon S. Keller, Bernd Weber, Peter N. Taylor, Marcus Kaiser

**Affiliations:** 1 Interdisciplinary Computing and Complex BioSystems, School of Computing Science, Newcastle University, Newcastle upon Tyne, United Kingdom; 2 Department of Biomedical Engineering, Korea University, Seoul, Republic of Korea; 3 Department of Brain Cognitive Sciences, Seoul National University, Seoul, Republic of Korea; 4 Department of Molecular and Clinical Pharmacology, Institute of Translational Medicine, University of Liverpool, Liverpool, United Kingdom; 5 Center for Economics and Neuroscience, University of Bonn, Bonn, Germany; 6 Department of Epileptology, University of Bonn, Bonn, Germany; 7 Institute of Neuroscience, Newcastle University, Newcastle upon Tyne, United Kingdom; Indiana University, UNITED STATES

## Abstract

Temporal lobe epilepsy (TLE) is a prevalent neurological disorder resulting in disruptive seizures. In the case of drug resistant epilepsy resective surgery is often considered. This is a procedure hampered by unpredictable success rates, with many patients continuing to have seizures even after surgery. In this study we apply a computational model of epilepsy to patient specific structural connectivity derived from diffusion tensor imaging (DTI) of 22 individuals with left TLE and 39 healthy controls. We validate the model by examining patient-control differences in simulated seizure onset time and network location. We then investigate the potential of the model for surgery prediction by performing *in silico* surgical resections, removing nodes from patient networks and comparing seizure likelihood post-surgery to pre-surgery simulations. We find that, first, patients tend to transit from non-epileptic to epileptic states more often than controls in the model. Second, regions in the left hemisphere (particularly within temporal and subcortical regions) that are known to be involved in TLE are the most frequent starting points for seizures in patients in the model. In addition, our analysis also implicates regions in the contralateral and frontal locations which may play a role in seizure spreading or surgery resistance. Finally, the model predicts that patient-specific surgery (resection areas chosen on an individual, model-prompted, basis and not following a predefined procedure) may lead to better outcomes than the currently used routine clinical procedure. Taken together this work provides a first step towards patient specific computational modelling of epilepsy surgery in order to inform treatment strategies in individuals.

## Author Summary

Temporal lobe epilepsy (TLE) is a disorder characterised by unpredictable seizures, where surgical removal of brain tissue is often the final treatment option. In roughly 30% of cases surgery procedures are unsuccessful at preventing future seizures. This paper shows the application of a computational model which uses patient derived brain connectivity to predict the success rates of surgery in people with TLE. We consider the brains of 22 patients as networks, with brain regions as nodes and the white matter connections between them as edges. The brain network is unique to each subject and produced from brain imaging scans of 22 patients and 39 controls. Seizures are simulated before and after surgery, where surgery in the model is the removal of nodes from the network. The model successfully identifies regions known to be involved in TLE, and its predicted success rates for surgery are close to the results found in reality. The model additionally provides patient specific recommendations for surgical procedures, which in simulations show improved results compared to standard surgery in every case. This is a first step towards designing personalised surgery procedures in order to improve surgery success rates.

## Introduction

Epilepsy is a spectrum of disorders characterised by recurrent seizures originating in the brain. Epileptic seizures are defined as spontaneous occurrences of signs and symptoms due to abnormal neuronal activity in the brain [1, 2]. Roughly 50 million people suffer from epilepsy [3] and in many cases it can be exceedingly debilitating. There are many types of epilepsy, classified by where in the brain seizures start or what the root cause is thought to be [4]. In this study we focus on the most common medically intractable form of epilepsy, temporal lobe epilepsy (TLE), characterised by a supposed focal origin of seizures in the temporal lobe.

Anti-convulsant drugs are the main method of treatment for epilepsy, however in about 30% of cases they are unsuccessful at preventing seizures. When drug therapies fail, surgery is an alternative treatment option for many patients. In TLE this involves the resection of regions of the temporal lobe assumed to be the source of the epileptic activity. Short term results show 53%-84% of patients achieving seizure freedom following surgery [5], however post-surgery longitudinal studies report only around 47%—65% of patients become seizure free following the resection of focal areas [6, 7].

Atrophy of focal brain areas in and around the temporal lobe is frequently found in people with TLE [8–14] using magnetic resonance imaging (MRI). However, recent suggestions are that TLE may involve areas far beyond the temoral lobe, forming a suggested epileptogenic network [15]. Diffusion weighted MRI (DW-MRI) allows for the inference of hypothesised white matter fibre tracts which connect brain areas [16, 17]. This can be used to create a patient specific network of structural brain connectivity between the aforementioned brain areas [18–23].

Epilepsy is a disorder which involves propagation of high amplitude oscillatory dynamics over large areas of brain tissue during seizures. This means that modelling a disorder such as epilepsy ideally requires a large scale model of the brain, with realistic connections through which the activity can spread. To this end the brain can be modelled as a network, with structurally distinct regions abstracted to single nodes and the connections between these discrete regions mapped through imaging techniques such as DW-MRI. Using such simplified brain networks, seizure initiation and spreading can be modelled using connectivity specific to individual patients [2, 24–26].

Computational modelling of epilepsy has been accomplished in various forms, though very few have used patient specific whole brain network data [27]. Most commonly neural mass models—simulated populations of connected neurons—have been used to produce or explore models of epileptogenesis [28–37]. Various mechanisms for seizure initiation have been proposed, and one of the most commonly used is bistability (see [2, 38] for reviews). This mechanism requires the system to have two stable states which coexist, a resting state (e.g. fixed point) and a state with seizure-like dynamics, (e.g. a high amplitude limit cycle). While in a parameter region where both these states are potentially available, the system is set to receive a noisy input such that when the noise passes a certain threshold the system transitions from one state to the other. A clear example of this in the context of epilepsy is the model described by Kalitzin *et al*. [39] and refined by Benjamin *et al*. [40], where one parameter controls the attraction of the system to the two stable states. This has been applied to network models to investigate the spread and likelihood of seizures dependent on connectivity [40, 41]. However, those studies did not use structural connectivity derived from DTI.

We aim to develop a framework for predicting surgical outcomes in patients following resective epilepsy surgery using a bistable model and DTI data obtained from patients with epilepsy. Following previous reported surgical success rates of around 50%-80% [5–7] we hypothesise that our model predictions shall also be in this range. Furthermore, we hypothesise that subject-specific surgery will give greater seizure reduction than that of standard resection procedures in our simulations.

## Results

As detailed in the modelling section, we applied a mathematical model to DW-MRI acquired patient specific connectivity networks and observed the results of simulations of regions of interest (ROI), or ‘node’, activity over time. Each ROI can transition (‘escape’) into a seizure state with a probability influenced by the region’s surface area, connectivity, and fluctuations in background noise. Escape times are taken to be representative of seizure likelihood, as a fast escape time means there would be more seizures on average for that node than for one with a slower escape time, i.e. a greater likelihood of a seizure occurring. Our analysis includes the simulations of 39 controls and 22 patients with left TLE.

### Model validation

**Fig 1:**
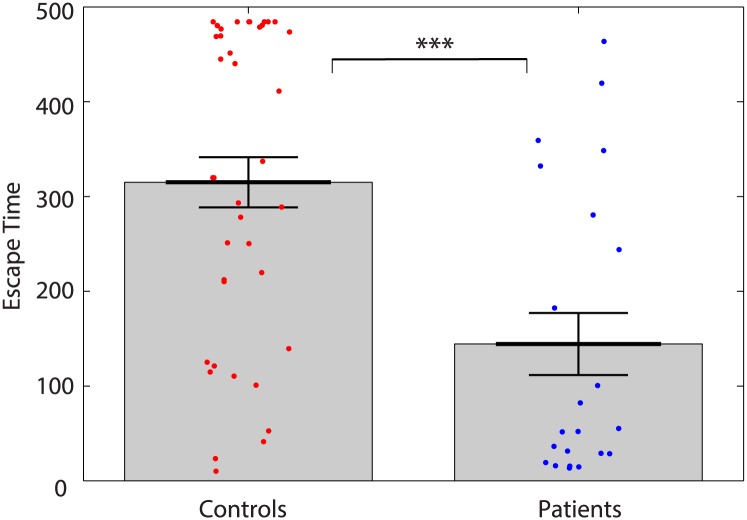
Patient’s nodes tend to escape to the seizure state faster than control’s. Comparison of patients and controls initial transition times into the seizure state. A seizure state was deemed to have occurred once three nodes had escaped, so the times here are when the third node hit the seizure state. Asterisks represent a significant difference found at *p* < 0.001 using a Mann-Whitney U test. Each dot represents the mean escape time for one individual subject over 100 iterations, grey bars reach the mean of each subject group, error bars show the standard error of the mean. Higher times indicate that the individual took longer to reach a seizure state.

In reality controls have a significantly lower likelihood of having seizures than patients. Fig 1 shows this, on average, to be the case in the model. A comparison of the times taken for the first three ROIs to escape from the resting state into the pseudo-seizure state for patients versus controls is shown in the figure, with a clear difference in distribution evident. The time taken for three nodes to escape was taken to be the seizure start time (‘escape time’) as we considered this to be evidence of the beginning of spreading of the seizure activity. The simulation was repeated 100 times with different noise seeds on each iteration, and the escape times were averaged over the 100 runs. These results are significant (*p* < 0.001) checked using a Mann-Whitney U test. These results also hold true for the first and second escape (S1 Fig).

**Fig 2:**
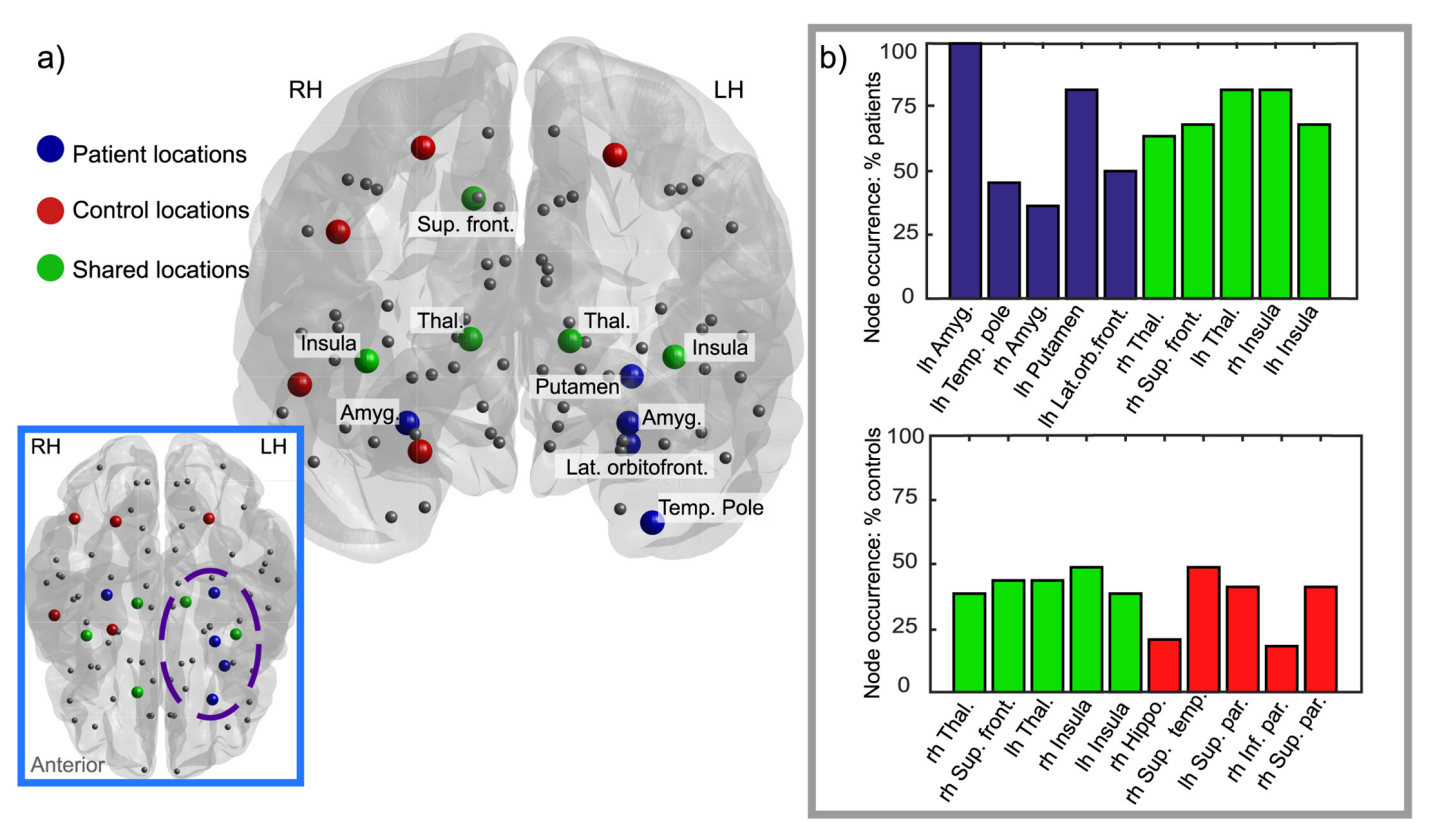
Patient-control ten earliest escape locations compared. Part a) shows the locations of the top ten most ‘seizure-prone’ nodes. These were found by taking the mode over the first three nodes to escape for each subject (split into patient and control groups) for all simulations without resections. The nodes represent locations in the brain network, and are coloured according to the group they escaped first in—blue for patients, red for controls and green for where the location was in the top ten to escape for both groups. Smaller grey circles show the locations of other nodes in the network which were not in either top ten. The main figure has location labels for patient areas, and the inset figure (transverse plane) has a dashed line encircling the main group of nodes for patients which are clustered in or around the temporal lobe. It is perhaps worth noting that while escape times are not apparent in this image controls nodes did take longer to escape than patients for all locations. Part b) shows the consistency of the top ten nodes compared for patients and controls, where patients show greater consistency for all the shared nodes and strikingly the left hemisphere amygdala escaped to the seizure state in every single patient at least once over 100 iterations.

Our second validation of the model examines where the initial escaping nodes are situated in the brain. In left TLE a seizure origin in the left temporal lobe would be expected for the model to match clinical observations. Fig 2a) shows the top ten escaping nodes, found by taking the mode for patient and control populations, in blue and red respectively. The five green nodes indicate locations in the top ten of both populations. S2 Table shows the top three nodes for every subject, from which the overall top ten were found, separately for patients and controls. While the red control escape locations are scattered with no discernible pattern, the blue patient escape locations are concentrated on the left hemisphere of the brain and predominantly in the temporal (temporal pole) or in subcortical (amygdala, putamen, thalamus) areas. Furthermore, there was a greater consistency of escaping nodes in patients than controls, with nodes in the top ten appearing 60.6% of the time. The left hemisphere amygdala was in the top three in 54% of the patients when taking the modes for each patient over all iterations, and occurred at least once in each patient top three over the 100 iterations. The consistency was reduced in controls, where nodes in the top ten appeared 30.7% of the time, with no one node occurring in more than 50% of controls over 100 iterations. Fig 2b) illustrates the difference with a bar plot comparison between patients and controls. The model therefore also reproduces a more stereotyped seizure pattern in patients, than in controls. This is in agreement with previous studies [25, 42].

### Model underpinnings

In order to pinpoint why we see the pattern of escaping nodes found in Fig 2 a number of network measures were tested. In the model the two major influences on node escape times are surface area and connectivity. Surface area alone does not correlate strongly with the pattern of fastest escaping nodes (see S3 Table) therefore we looked at the connectivity to see whether there were any clear markers of seizure prone nodes from connectivity measures. One possibility was that hub nodes could prove to be more seizure prone, as there are ROIs with a large number of incoming connections and as such may receive large inputs pushing them closer to the seizure threshold. Additionally the high number of connections means they are more likely to receive input from a node which has already hit a seizure state and are then liable to be pushed over the seizure threshold themselves by the pathological input. We predicted that the nodes which appear as fast escapers for both patients and controls may be natural hubs, explaining their appearance in Fig 2.

**Fig 3:**
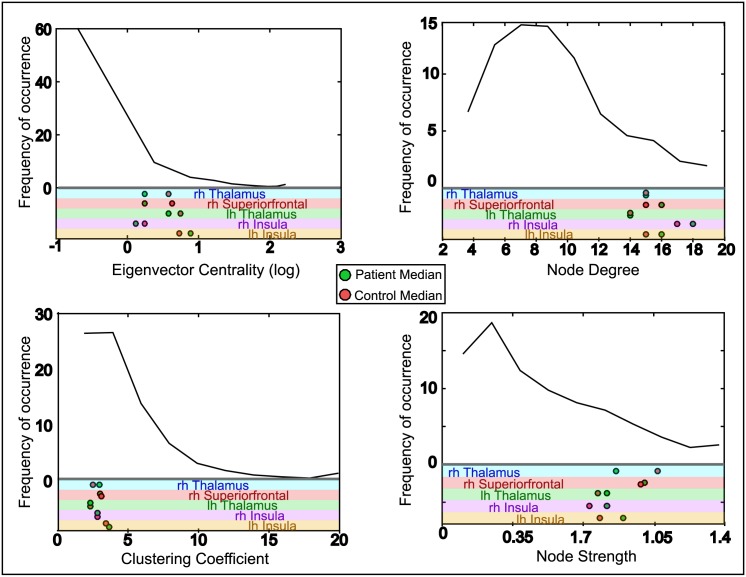
Commonly escaping nodes shared between patients and controls are network hubs. Five of the most commonly escaping nodes in patients and controls occurred in both groups, and different node measures are compared for these nodes here. For each network measure in the figure the distribution for all nodes in all subjects is shown in the top section of the plots. The dots in the lower section reveal where in this distribution the shared nodes occur for the given measure. These locations are shown separately for the ROI in patients and controls (coloured green and red respectively) displaying the median value for the groups. The high node strength, degree and eigenvector centrality values for the shared nodes with a low clustering coefficient support the idea that these regions may be network hubs.

**Fig 4:**
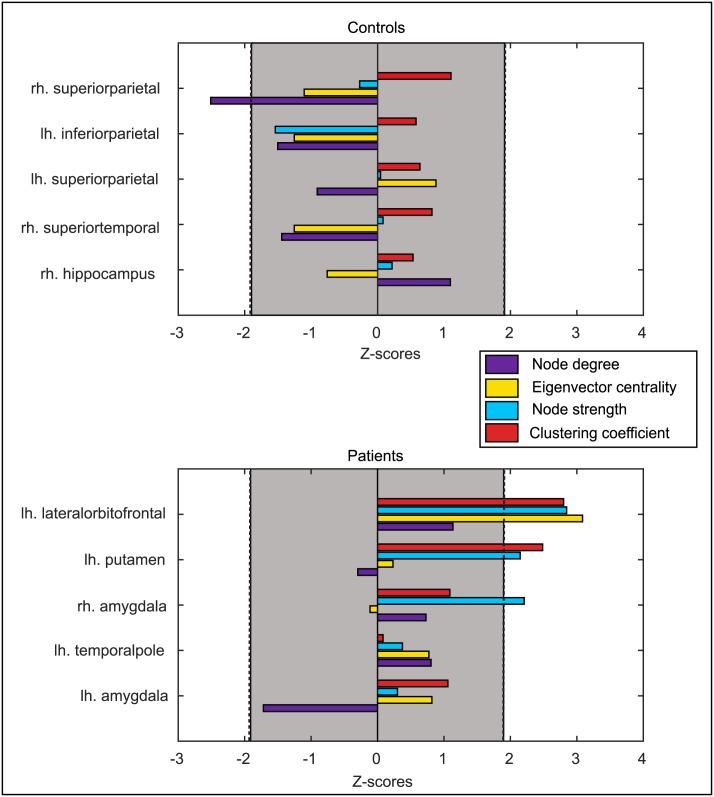
Commonly escaping nodes which differ between patients and controls compared. As these nodes have different escape time patterns in patients and controls we checked for network differences between the two groups. Z scores were found using a Mann-Whitney U test comparing network measures for the unshared nodes between patients and controls. A negative value means that the node score was lower in patients than in controls, and a positive z score means the node score was higher in patients than in controls. There are no clear differences which implies more complex origins for the different escape time results.

Fig 3 focuses on the five nodes which occurred in the top ten escaping nodes of both patients and controls. The node measures for the shared five are displayed beneath a map of the distribution of that node measure for all subjects. The results do support the idea that the shared nodes are liable to be network hubs, showing a low clustering coefficient value but much higher node strength and degree values than most ROIs. Fig 4 shows the remaining unshared nodes in the top ten for patients and controls compared using a Mann-Whitney U test for the ROI in patients against the same region in controls for given network measures. Because these differ between groups we looked for evidence that there were differences in the node network properties between the groups. However there were no clear trends to indicate that any one network measure could account for these nodes being in the top ten.

In summary, the rapid escape time of some nodes can be attributed to shared properties of being network hubs. However, the complex interplay of connectivity, surface area, variations in streamline length (delays) and nonlinearities in the model, leads to highly nontrivial outcomes in the simulations.

### Model prediction

**Fig 5:**
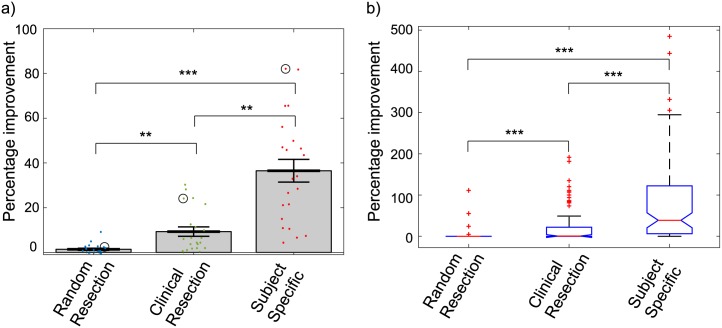
Simulated surgery results comparisons. For every patient three different methods of resection were used and then the escape times were compared for each patient pre and post surgery (surgery simulation results compared to results from simulations where no surgery was carried out). Plot **(a)** shows the mean percentage improvement in escape time for each patient by resection method, where escape time was taken to be the time by which 3 nodes had hit a seizure state. Grey bars reach the mean of each subject group, error bars show the standard error of the mean. The circled values are the means for the patient shown in plot (b). A single patient representative example is shown in plot **(b)**, with the percentage improvements found for each simulation plotted and compared by resection technique. Stars represent the significance level, 1 star for significance at *p* < 0.05, 2 stars for *p* < 0.01 and 3 stars for *p* < 0.001. A Mann-Whitney U test with Bonferroni correction was carried out to find the p-values following significant results found from Kruskal-Wallis tests.

Here we test the impact of removing 1) randomly selected adjacent nodes, 2) nodes routinely removed clinically (amygdala, hippocampus and parahippocampal gyrus) and 3) optimised patient specific nodes. It is worth noting here that the patient specific resections removed the top three most seizure prone nodes for each individual patient. This did not account for whether the chosen ROIs would be practical to remove in an actual surgery procedure. Fig 5(a) shows a comparison of the simulation results when using different resection methods. The percentage improvements were found for each resection technique, and the mean improvement for each patient is plotted in this figure. There is a clear increase in improvement from random resections to clinical, and then again to the subject specific resections. Asterisks indicate that the distributions of improvement times are significantly different between techniques. The simulations were repeated over 100 iterations with differing noise seeds, and comparisons of the escape times were patient by patient. Subject specific resections removed the top 3 fastest escaping nodes for each patient. (S2 Table shows the top 3 escaping nodes for each patient).

Fig 5(b) shows a comparison of improvements in escape time for different resection methods for a single subject. In this case the simulated clinical resection was successful, producing significant improvements. The subject specific resection was even more successful, significantly reducing seizure likelihood compared to unresected and also the clinical resections. When looking directly at escape times the difference between the unresected and random resection simulations was insignificant at *p* < 0.05, both for the patient singled out in Fig 5(b) and when comparing all patient simulations together.

Finally Table 1 compares the percentage of patients who show significant improvements in our model following resection of clinically removed areas with established literature on surgery success. Simulation results were found using a paired t-test comparing before and after escape times for each patient over all iterations, with a significance level of *p* < 0.05. The removal of clinically resected regions in our model produced significant improvements in 72.7% of patients, and the subject specific resections showed improvements in 100% of cases (also included in the table). There is a wide range of reported success rates in literature, partially due to the time scales of studies (ranging from 1 year after surgery to 18) and due to different definitions of significant improvement. Despite the variability the model results fit squarely in the middle of the reported range, while the subject specific results show significant improvements in every case.

**Table 1:** Percentage of subjects with significant improvements following surgery. A comparison of the model generated success rate with reports in literature. In studies there have not been any patient specific procedures reported to compare, so these reports only include widely used clinical procedures including the amygdalohippocampectomy. The wide ranges in some reports are due to studies grouping patients by different criteria such as lateralisation of atrophy, or in other cases due to longitudinal studies measuring improvements at several different time points after the surgery. There is a general trend of decreased improvement the longer the time period after surgery, reducing the reported successes.

Source	Clinical (%)	Patient Specific (%)
Model simulations	72.7	100
Spencer *et al*. 2008 [5]	53–84	-
Hemb *et al*. 2013 [6]	62–65	-
de Tisi *et al*. 2011 [7]	52–47	-
Arruda *et al*. 1996 [43]	50–93.6	-

## Discussion

In this work we have presented three key findings. First, we have demonstrated how the combination of subject specific data and a nonlinear computational model can lead to successful identification of TLE associated nodes in patients. Control subjects show few similarities in identified nodes indicating clear differences in network organisation (Fig 2). Secondly, we have shown in the model, in agreement with with clinical observations [6], that selective amygdalohippocampectomy can result in reduced seizure likelihood in 72.7% of patients, which is within the range reported in the literature for patients with similar conditions (Fig 5 and Table 1). Third, we suggest that patient-specific operations (where different regions are selected for different patients) will result in a better outcome. Furthermore, to our knowledge, this is the first study to combine computational modelling of brain dynamics with diffusion MRI based connectivity data from patients with temporal lobe epilepsy.

### Model validation

The model validation Figs 1 and 2 demonstrate results in line with clinical observations—a higher propensity for seizures in patients and the spatial origin of seizure activity in the ipsilateral temporal lobe. Indeed, validation of any model should always be the crucial first step. One aspect the model does not accurately capture is that healthy controls still have seizures in the model. Healthy controls *can* have a seizure—they have a propensity to seize, however, this would typically be a result of an external factor such as trauma and therefore may be mechanistically different. It is plausible to suggest that there may be other mechanisms and contributors to seizure transition (besides those included here) which may be important to improving the model, especially when considering healthy controls.

Another aspect that the model successfully validated was the spatial profile and consistency (stereopy) of the ‘escaping’ nodes. Several areas located in the ipsilateral temporal lobe and subcortical areas were involved in agreement with experimental / clinical observations [20, 44, 45]. Four areas not in the left hemisphere were involved—the amygdala, insula, thalamus and the superior frontal region. Contralateral abnormalities have been noted in TLE before [20, 46], particularly in temporal lobe areas [15]. It has been speculated that this could be indicative of compensatory mechanisms of some form, as increased contralateral functional connectivity has been previously noted in epilepsy patients [47]. If there is a structural correlate for this potential compensatory connectivity increase then a larger number of connections may put these regions more at risk of seizures in the model, for the same reasons as highly connected hub nodes are vulnerable. A high number of inbound connections increases the likelihood of receiving abnormal input from a seizing node, and increases the input received pushing the node closer to the seizure threshold. The involvement of the amygdala, thalamus and insula in TLE has long been known [48–50]. Interhemispheric connectivity between the mirrored regions in both hemispheres may be the cause of the seizure vulnerability of the contralateral amygdala, thalamus and insula. Abnormal activity from the ipsilateral hemispheric counterparts may spread through interhemispheric connections. The superior frontal region involvement is less intuitive, and the lateral orbitofrontal region is also a rapidly escaping node located in the frontal lobe. Abnormalities in frontal lobe areas have been documented in TLE [51–53], with frontal regions frequently involved in seizure propagation, especially the orbitofrontal cortex [54]. This may provide an explanation for the susceptibility of these nodes to seizures in our model.

### Model underpinnings

Network measures were tested for the most frequently escaping nodes in an attempt to distinguish further the influences behind the seizure susceptibility. Five of the top ten escaping nodes are consistent in both patients and controls, and were found to exhibit properties of network hubs, as shown in Fig 3. These were the thalamus (bilaterally), insula (bilaterally) and the contralateral superior frontal area. The thalamus and insula are already known to be highly connected and hub-like in the general population [55]. The high node strength and centrality measures of these regions make them both good candidates for network hubs [56], and also more likely to propagate abnormal activity. Having a greater number of incoming connections puts hubs at greater risk of receiving input from a seizing area, and it follows that they would also propagate the abnormal activity to a large number of regions they connect to. For the remaining five nodes that differ for patients and controls seizure susceptibility is less clear. There is no defining contribution from either topological (see Fig 4) or spatial (e.g. surface area, fibre tract length—see S3 Table) features, although the lambda parameter derived from surface area does have an effect on the patient nodes. Previous studies have looked for network differences in TLE, finding changes in clustering coefficient in some areas, small world-ness and network efficiency when comparing to controls [19–21], summarised in [27]. However a recent study into the same dataset we use here found that spatial measures show far greater changes in TLE than network measures [8]. Whilst it is possible that a feature may have been missed, it seems that a combination of multiple interacting factors is most likely at play in determining which ROIs are most seizure prone in the model.

### Model prediction

The *in silico* resection experiments showed improvements for the clinically removed nodes to a similar extent to results found in reality, with 72% of patients (16 out of 22) showing significant improvements (*p* < 0.05) in seizure likelihood after simulated surgery. In this instance statistically significant improvements were taken to be analogous to successful surgery procedures. In many surgery cases the patients are not completely seizure free but they do see some reduction in seizure likelihood or intensity. This is a phenomenon which is reflected in some ways in our results, with an overall skew of improvement, but not in all cases statistically significant.

The random resection experiments showed that the clinical resection results are not simply due to the removal of any 3 nodes from the network. There was some improvement for the random resections, although this was only to a statistically significant extent in one or two cases (depending on the nodes removed), much less than for clinical resections.

The personalised resections showed by far the most success at reducing seizure likelihood. These were based on the top three fastest escaping nodes for each patient. The ipsilateral hippocampus did not appear in any of the top escaping node vectors for patients. This is surprising given the well established phenomenon of hippocampal sclerosis in TLE [57, 58], and the fact that it is commonly removed in resection surgery. In one of the random node tests the ipsilateral hippocampus was included in the 3 randomly selected regions to be removed, but this did not show any greater improvement than other random node assortments. The reasons for this unexpected result are unclear, and possibly due to the difficulty of parcelating subcortical structures in Freesurfer [59]. Although the outputs were checked visually [8] this may have impacted results. If the hippocampus had a significant impact on ictal genesis then it would seem logical that removing it would have a greater impact on seizure likelihood.

As well as the ipsilateral temporal pole, the thalamus and insula both appeared commonly in the sets of earliest escaping nodes. The insula has been linked to TLE, with studies suggesting that a number of patients with unsuccessful surgical outcomes may have an ictal focus in the insula, hence the lack of results from temporal lobe resections [49, 60–62]. The thalamus may similarly play a role in surgery resistent TLE [63, 64], and has been indicated to have an important role in the propagation and generation of TLE seizures [50, 65]. The thalamus has been found to exhibit abnormalities in TLE [66] with cell atrophy and reduced surface area [67]. However the thalamus is crucial for sensory-motor functions and therefore not an option for resection. We speculate that the partial disconnection of the ipsilateral thalamus (rather than the complete resection) may lead to improved clinical results.

### Surgery prediction in the context of other works

This is not the first or only attempt to try and accomplish prediction of the affects of neurosurgical procedures. Imaging studies have been used to predicted how TLE surgery effects memory and cognitive ability in individuals [68]. While computer modelling has been applied to measure the effects of *in silico* lesions on connectivity networks [69, 70], none have used DTI derived networks of people with TLE. One study by Honey *et al*. [71] used computational modelling of macaque connectivity with Kuramoto oscillators and a neural mass model to examine how region synchrony and interaction differed after lesions. However, that study did not look specifically at effects on seizure activity.

Recent modelling work has attempted to better characterise and identify epileptogenic areas [28, 34] with mention of the potential effects of surgical intervention dependent on tissue characteristics. Accurately identifying regions of seizure initiation is clearly an important consideration in surgery planning and prediction. These studies did not use whole brain connectivity, but rather a model representing a subset of cortical areas. Another recent work, incorporating patient derived connectivity, found encouraging results in simulations of stimulation applied to abate seizures [26]. This is an intriguing alternative to surgery and one which can also benefit from subject-specific modelling work.

A study by Sinha *et al* [72] is closer to the aims and findings of this current project. Using ECoG data to derive connectivity from functional synchrony the study applied a very similar model to the one we have used here, without the incorporated surface area data. The model predicted areas for surgery removal and tested *in silico* resections against *in silico* randomised resection trials. The predicted regions for removal overlapped with clinically chosen resection areas (identified by the ECoG testing among other clinical evaluations) in 5 out of 6 cases. The model also predicted additional removal sites not identified by clinical tests. Those predictions were not experimentally validated, but this is another strong example of how computational modelling has the potential to be useful for surgery prediction, not limited to one method of connectivity data acquisition.

### Limitations of the study

The connectivity is assumed here to be a good and accurate representation of the white matter tracts in the individual’s brains. However, there are restrictions of resolution and noise in this imaging technique and the connectivity matrices produced may not always be fully accurate [73]. A key limitation of diffusion inferred networks is that they are bi-directional. This assumption of bi-directionality may be inaccurate, with one recent study into the Macaque brain finding a higher incidence of unidirectional links than anticipated [74]. Directionality has been shown to play a key role in the dynamics of a system and therefore should be considered a limitation of this and all diffusion based connectivity studies [75]. However the assumption of bi-directionality has also been defended in other studies which claim that the majority of brain connections do exhibit bi-directionality [76]. There is currently no better alternative for imaging structural connectivity *in vivo*, but this is still a potential limitation. One possible solution to this, which could especially be done in the case of epilepsy patients, would be to map the directionality through active stimulation [77, 78].

Another limitation is the use of deterministic tractography in this study. Crossing fibres are not well resolved in deterministic approaches, which can lead to inaccurate representations of brain connectivity [79].

This is not an optimal protocol, and probabilistic algorithms would likely improve the accuracy of the connectivity matrices by resolving the crossing fibre problem [80]. However, there have been studies which have found that deterministic tractography can succeed at capturing key features of the connectome and thus can provide useful information [81–84]. Additionally deterministic tractography can have greater success at characterising long range connections [85], which are important when attempting to characterise seizure spreading patterns. Deterministic and probabilistic tractography capture graph theory metrics similarly, share common features [86], and can be remarkably reproducible [87]. That being said, as probabilistic tractography has been found to be the superior method in terms of reproducibility and crossing fibre resolution giving a more reliable connectome [80, 86, 88] it may therefore be beneficial to rerun simulations using a probabilistic algorithm.

In addition to the innate issues with DTI, there is also the question of how to decide on the brain atlas to use for parcellating into regions, and the choice of weighting used for edges. The dataset used here split the brain into 82 different regions. There is no gold standard for choosing the number of regions to use, and this choice can make a difference to the network’s properties [89]. Comparisons have found that network organizational principles seem to remain independent of the parcellation scheme, but quantified measures do change [90]. A future direction would be to replicate this study with an alternative, functionally defined atlas (e.g. [91]). However, functional parcellations often don’t include subcortical areas and are based on functional observations in healthy subjects. The possibility of changes in underlying processes due to epileptic activity may make existing functional schemes inaccurate for patients. Applying alternative anatomical atlases would also be worthwhile further work. We anticipate, though it should be tested, that results shown here would be reproducible with other atlases at similar scales. This prediction is based on comparisons of our results with previous studies [92] including work simulating epilepsy dynamics with human connectome data using different atlases (AAL and Desikan) [24–26]. Choosing the weighting of edges is another factor to take into account. In this instance the dataset weighted edges by the number of streamlines identified, however other options are available such as fractional anisotropy, which weights edges according to the degree of anisotropy in voxels between regions, thought to give an idea of fibre myelination or integrity [93, 94].

Another criticism of the model could be found in the level of abstraction used. Limitations in the computational resources available inevitably leads to a trade off when abstracting a model. There may be a danger of losing information from the system which is present in biological reality, for example meso- and micro-scale connectivity were not modelled here, yet may be important [95]. As a model to assess the impact of global connectivity on TLE, having too many other interacting factors would make it more difficult to identify the contribution of heterogeneous networks and a danger of over-fitting the parameters may occur. However, as a model for surgery prediction perhaps more biological detail would be useful to improve accuracy when looking to match *in vivo* results more closely. The compromise between simulation speed and biological detail can be difficult. Even though this model is phenomenological, the simple co-dependence on region surface area and connectivity makes it computationally feasible to simulate while producing encouraging results.

### Future work and improvements

It is necessary to validate the model prediction of subject specific surgical success with patient outcomes if the model will ever be useful clinically. Diffusion imaging is not routinely done and it is extremely difficult to obtain data for a significant number of subjects with follow-up metadata of surgical outcome. One of the key advantages of our study is that we have a fairly homogeneous subject group (all patients with left TLE), however, statistical power becomes an issue with a total sample size of only 22 subjects. We anticipate that in the future a multi-site study with large cohorts will be required. With that we hope to enable better guidance for epilepsy surgery.

## Methods

### Dataset

We collected data from 22 individuals with left mesial TLE showing unilateral hippocampal sclerosis and 39 age-matched controls. Written informed consent was obtained, signed by all participants, and conformed to local ethics requirements. IRB approval (032/08) was given by the ethical review board of the medical faculty of Bonn. None of the individuals with mTLE had undergone any previous neurosurgery. T1 weighted MRI scans and diffusion tensor imaging (DTI) data were obtained using a 3 Tesla scanner, a Siemens MAGNETOM TrioTim syngo (Erlangen, Germany). The T1 images were obtained using 1mm isovoxel, TR = 2500ms and TE = 3.5ms. The DTI data used 2mm isovoxel, TR = 10,000ms, TE = 91ms and 64 diffusion directions, b-factor 1000s mm^−2^ and 12 b0 images. In both cases FoV was 256mm.

**Fig 6:**
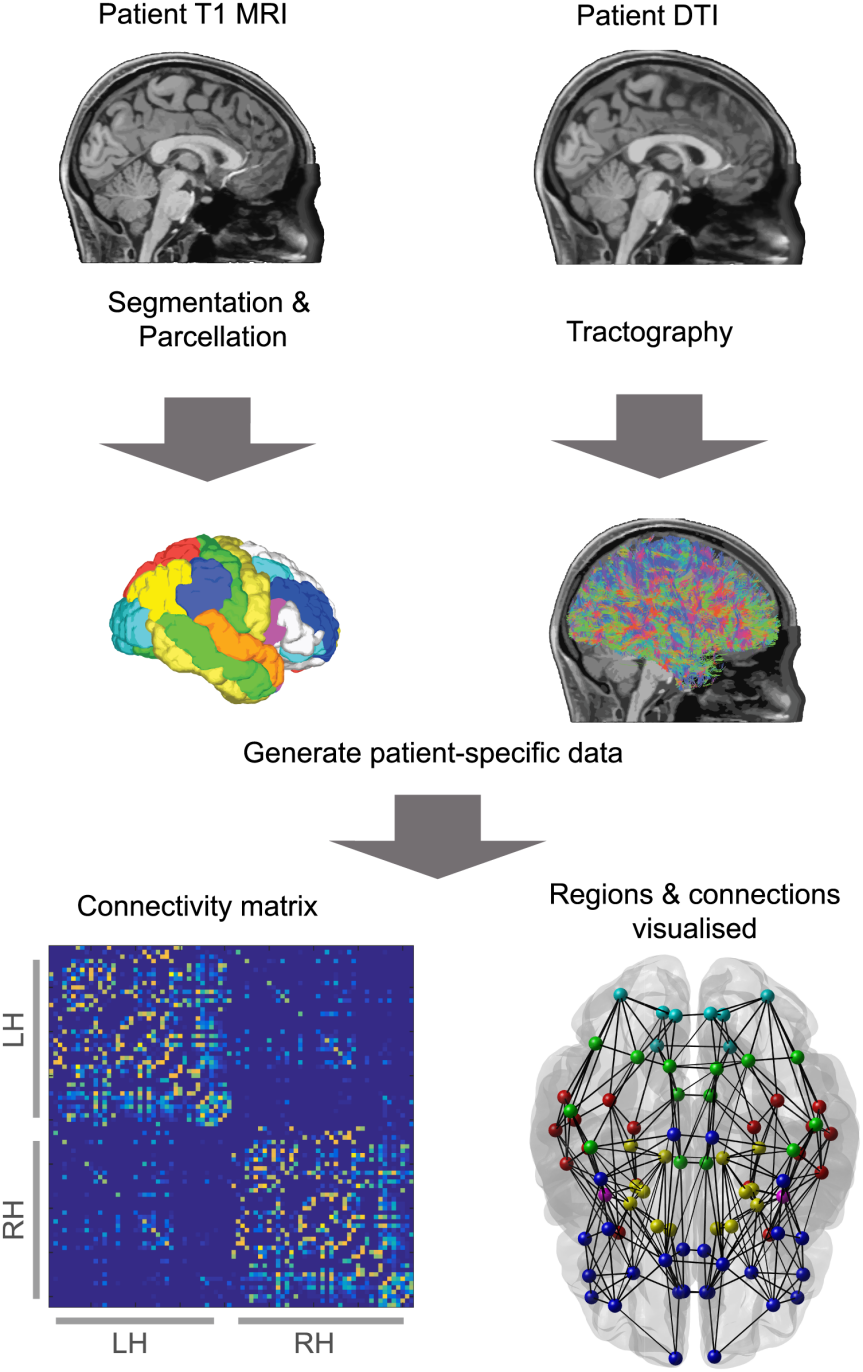
The creation of a connectome. A diagrammatic representation of the connectome creation process, combining information from T1 MRI and DTI to create a network formed from brain regions connected by white matter fibres. In this case parcellation of the brain into different regions split the grey matter into 82 ROI, including 14 subcortical regions. The bottom images show the resulting connectivity matrix (left) of the connectome and the connectome visualised as nodes and edges overlaid on a brain shadow (right), where colours denote lobe areas.

**Fig 7:**
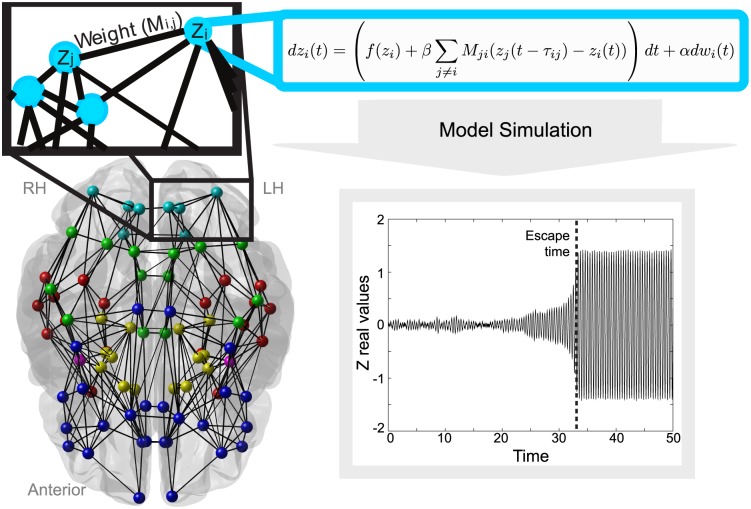
Connectivity, node placement and how this relates to the mathematical model. Connectivity is shown where connections exist in 60% or more of subjects, for visualisation only—all connections were included on a subject specific basis for simulations. Nodes and connections are overlaid on a 3D brain image to show the rough positioning of the nodes and the distance of connections between them. The nodes are the positions of ROI, with colours corresponding to different lobes, and the black lines show the connections between different regions. The zoomed in top left graphic shows how connections between nodes are weighted, and the blue box shows how the activity change of one node is found using the model. The bottom right figure shows a time series of the model simulation. The model begins in the background state with low amplitude oscillations and transits to a high amplitude seizure-like state at approximately t = 14, indicated by the dashed line. In this exemplary case the escape time would be taken as 14 for the node in this simulation.

Connectome creation from the acquired data is summarised in Fig 6, while Fig 7 shows nodes and connections imaged in pseudo-3D space averaged over all subjects, and indicates how this is then used to generate simulations of activity for each node. Firstly FreeSurfer [96] was used to obtain surface meshes of gray and white matter boundries from the MRI data, and to parcellate the brain into regions of interest (ROI) based on the Desikan atlas [97, 98]. There were 82 ROI included, spanning cortical and subcortical regions (subcortical regions included the Nucleus accumbens, Amygdala, Caudate, Hippocampus, Pallidum, Putamen and the Thalamus). Streamline tractography was obtained from DTI images using the Fiber Assignment by Continuous Tracking (FACT) algorithm [99] through the Diffusion toolkit along with TrackVis [100]. First we performed eddy-correction of the image by applying an affine transform of each diffusion volume to the b0 volume and rotating b-vectors using FSL toolbox (FSL, http://www.fmrib.ox.ac.uk/fsl/). After the diffusion tensor and its eigenvector was estimated for every voxel, we applied a deterministic tractography algorithm [99] initiating a single streamline from the center of each voxel. Tracking was stopped when the angle change was too large (35 degree of angle threshold) or when tracking reached a voxel with a fractional anisotropy value of less than 0.2 [84]. For further details see [81].

The centre coordinates of each voxel were the start of a single streamline, the total number of streamlines never exceeded the number of seed voxels. The number of connecting streamlines were used to determine the connectivity matrix (*S*), as the streamline count has recently been confirmed to provide a realistic estimate of white matter pathway projection strength [101]. Distance matrices were also constructed using the mean fibre length of the streamlines connecting each pair of ROIs. The surface area of each ROI was found using FreeSurfer for cortical regions and for subcortical areas by computing the interface area to the white matter in T1 space [102].

### Model

We apply a mathematical model to simulate activity of ROI in a network formed from acquired DTI data. The network connectivity matrix, *M*, is determined by the patient specific weighted connectivity produced in connectome creation as detailed in the previous section. The connection weights were derived from the streamline counts between ROIs and were normalised as shown in Eq (2), where *S* is the matrix of streamline counts for an individual subject.

$$\begin{array}{c} M=\frac{log(S)}{max(log(S))} \end{array}$$ (1)

The model used in this paper is adapted from Benjamin et al.’s 2012 paper [40] which is derived from the work of Kalitzin [39] and has been previously used for simulating epilepsy surgery [72]. To summarise, this model allows for noise dependent transitions from low to high amplitude oscillations while in a bistable parameter region. The activation level of the *i*th node, *z*_*i*_, is determined by Eq (2) below:

$$\begin{array}{c} dz_{i}\left(t\right)=(f\left(z_{i}\right)+\beta \sum_{j\neq i}M_{ji}\left(z_{j}\left(t-\tau_{ij}\right)-z_{i}\left(t\right)\right))dt+\alpha dw_{i}\left(t\right) \end{array}$$ (2)

where *f*(*z*_*i*_) is a function of *z*_*i*_ described in Eq (3), *w*_*i*_(*t*) is a normally distributed noise term, *α* scales the noise, *β* scales the connectivity matrix *M*. *M*_*i*,*j*_ is the connection strength between the *i*th and *j*th nodes, and *τ*_*i*,*j*_ is the delay between them. *f*(*z*_*i*_) is defined by:

$$\begin{array}{c} f\left(z_{i}\right)=\left(\lambda_{i}-1+\hat{i}\omega \right)z_{i}+2z_{i}|z_{i}{|}^{2}-z_{i}{\left|z_{i}\right|}^{4} \end{array}$$ (3)

Where *z* is complex, *λ*_*i*_ controls basin of attraction to the background and seizure-like states, *ω* controls frequency of oscillations and $\hat{i}$ is imaginary.

Delays between areas (*τ*_*i*,*j*_) are incorporated into the model as proportional to the mean streamline length, grouped into bins, and use a biologically plausible propagation speed of 7 metres per second [25, 103]. S2 Fig explores a comparison between mean and median fibre lengths as a justification for this approach.

The parameter *λ*_*i*_ is derived from the difference (*D*_*i*_) of the specific subject’s region surface area from the control group distribution of region surface areas for the region *i*, as described in Eq (4):

$$\begin{array}{c} \lambda_{i}=\frac{-D_{i}}{\psi }+0.5 \end{array}$$ (4)

The difference (*D*_*i*_) is a measure of how many standard deviations away from the control group distribution the subject is, found through standard scores with tie-adjustment and continuity correction. In our case a very negative difference indicates a reduced surface area in region *i* compared to the control group, and *vice-versa*. In the controls a leave-one-out method was used to quantify how far each control was from the remaining controls. The *ψ* is set to 15, giving a wide spread for *λ* whilst ensuring 0 < *λ* < 1, i.e the bifurcations to monostability for all subjects.

The *ω* value controls the frequency of oscillations, and in all results detailed here this was set to a value of 15 to produce an oscillation frequency of 3Hz in the seizure state, which is typically observed in the intracranial electrocorticographic recordings of many patients with TLE [104]. The randomised noise *w*(*t*) is a complex Wiener process approximated by the Matlab function randn, generating normally distributed random numbers; this noise is scaled by *α*. Matlab was used to implement the model, and a fixed step Euler-Maruyama solver was employed to find solutions to the equations. Parameter *β* was fixed at a value of 0.01 for all subjects, and delays were found using the mean fibre lengths. The parameter *α*, which scales the amplitude of the noise, was set to a fixed value of 0.05 in order to allow a fair comparison of escape times without biasing by external factors, i.e. the only parameters influencing comparisons are those derived from the subject specific MRI (*M*, *λ*, *τ*).

In line with previous studies [40, 72] we use ‘escape time’ as a measure of epileptogenecity. Simulations are performed with the initial conditions placing all nodes in the background state, then upon simulation we measure how long it takes for each node to transit to the seizure state. This is determined by measuring the Euclidean distance *E*_*i*_ from the fixed point (0,0), over time. Only when *E*_*i*_ > 1 is the system considered to be in a seizure state. Thus, the escape time of node *i* is *min*(*t*(*E*_*i*_ > 1)). This is illustrated in Fig 7 (lower right panel).

### Validation of the model

To test the performance of the model we ran simulations for both controls and patients over 100 iterations with changing noise seeds. The two groups were compared to identify any differences in either the node escape times or in the locations of commonly escaping nodes. In addition to comparing the time taken for the first node to escape we also ran simulations until three nodes had escaped in succession, taking this as an indicator that the activity had begun to propagate.

### Simulation of surgery

We simulated three different conditions of surgery: 1) random resection, 2) clinical resection, and 3) patient-specific resection. Simulating surgery was achieved by setting inputs and outputs to and from a resected node to zero values, effectively isolating the node from the network.

1. Random resections removed three nodes from the network, the first chosen at random and then its two nearest neighbours with connection weights greater than the average were also removed, in order to remove a set of nodes with similar characteristics to those removed in clinical resections. These resections serve as a benchmark to which other resective techniques can be compared.
2. Clinical resections removed three regions most commonly resected in an amygdalohippocampectomy procedure (ipsilateral hippocampus, amygdala and parahippocampal gyrus).
3. For subject specific optimised resections, the top three fastest escaping nodes were found for every patient and subsequently removed (see S2 Table for a list of the top three nodes for each patient). These top escaping nodes were different for every patient, and in many cases the vector of resected nodes included one or more of the clinically removed areas.

## Supporting Information

S1 Fig Patient and Control escape time comparison, first and second nodes to escape. Similar to Fig 1, controls and patients show different distributions of escape times. Part a) shows the times for the first nodes to escape, and part b) plots the times taken for the second nodes to escape in each subject.
(EPS)

S2 Fig Mean streamline length is similar to median streamline length between regions. Exemplary connections between brain areas demonstrating similarity of streamline length measures. a) Streamlines connecting the left (purple) and right (red) superior frontal areas. Streamlines are coloured according to their direction. b) Histogram of the lengths of the streamlines shown in a). c) Streamlines connecting the precentral (yellow) and postcentral (red) gyri in the right hemisphere. Transparency of the gyri enables visualisation of the streamlines. d) Histogram of the lengths of the streamlines shown in c).
(EPS)

S1 Table The numerical codes for different regions. This table shows which numbers correspond to which brain region. ‘lh’ is short for left hemisphere, (nodes 1 to 41) while ‘rh’ is short for right hemisphere (nodes 42 to 82).
(PDF)

S2 Table Top escaping nodes for all subjects. This table shows a list of the 3 nodes which were consistently the earliest to escape to a seizure state for each individual in patients and controls, ranked in the order of number of appearances. It additionally includes patient age and gender information, where M indicates a male and F female. The numerical code for the node regions is explained in the previous table. Where there is a NaN (‘Not a Number’) label instead of a number there were only one or two nodes which were consistently the earliest to escape.
(PDF)

S3 Table A table of further node measures for the fastest escaping nodes of patients and controls. Fibre length measures and surface areas were checked and documented to look for any evidence of influence. The table shows z scores found from mean fibre lengths normalised by maximum fibre length and z scores from surface area data. These standard scores show the deviation from the main distribution and were found for the mean lengths of fibres connected to the fastest nodes, as well as for the surface areas of the fastest nodes. These measures were checked for all subjects and also within subject groupings of patients and controls.
(PDF)

S4 Table Further patient information. Additional information recorded about the patients included in this study. FS in the Early insult column is short for Febrile Seizures, and the seizure types recorded are simple partial seizures (SPS), complex partial seizures (CPS) and secondary generalised tonic clonic seizures (SGTCS).
(PDF)
