# Supplementary material for: Predicting Surgery Targets in Temporal Lobe Epilepsy through Structural Connectome Based Simulations
Source: PLoS Comput Biol. 2015 Dec 10;11(12):e1004642. doi: 10.1371/journal.pcbi.1004642 (PMC4675531; doi:10.1371/journal.pcbi.1004642)
Supplement: S1 Table — This table shows which numbers correspond to which brain region. ‘lh’ is short for left hemisphere, (nodes 1 to 41) while ‘rh’ is short for right hemisphere (nodes 42 to 82). (PDF) [file pcbi.1004642.s003.pdf]

1	lh.bankssts	42	rh.bankssts
2	lh.caudalanteriorcingulate	43	rh.caudalanteriorcingulate
3	lh.caudalmiddlefrontal	44	rh.caudalmiddlefrontal
4	lh.cuneus	45	rh.cuneus
5	lh.entorhinal	46	rh.entorhinal
6	lh.fusiform	47	rh.fusiform
7	lh.inferiorparietal	48	rh.inferiorparietal
8	lh.inferiortemporal	49	rh.inferiortemporal
9	lh.isthmuscingulate	50	rh.isthmuscingulate
10	lh.lateraloccipital	51	rh.lateraloccipital
11	lh.lateralorbitofrontal	52	rh.lateralorbitofrontal
12	lh.lingual	53	rh.lingual
13	lh.medialorbitofrontal	54	rh.medialorbitofrontal
14	lh.middletemporal	55	rh.middletemporal
15	lh.parahippocampal	56	rh.parahippocampal
16	lh.paracentral	57	rh.paracentral
17	lh.parsopercularis	58	rh.parsopercularis
18	lh.parsorbitalis	59	rh.parsorbitalis
19	lh.parstriangularis	60	rh.parstriangularis
20	lh.pericalcarine	61	rh.pericalcarine
21	lh.postcentral	62	rh.postcentral
22	lh.posteriorcingulate	63	rh.posteriorcingulate
23	lh.precentral	64	rh.precentral
24	lh.precuneus	65	rh.precuneus
25	lh.rostralanteriorcingulate	66	rh.rostralanteriorcingulate
26	lh.rostralmiddlefrontal	67	rh.rostralmiddlefrontal
27	lh.superiorfrontal	68	rh.superiorfrontal
28	lh.superiorparietal	69	rh.superiorparietal
29	lh.superiortemporal	70	rh.superiortemporal
30	lh.supramarginal	71	rh.supramarginal
31	lh.frontalpole	72	rh.frontalpole
32	lh.temporalpole	73	rh.temporalpole
33	lh.transversetemporal	74	rh.transversetemporal
34	lh.insula	75	rh.insula
35	lh.thalamus	76	rh.thalamus
36	lh.caudate	77	rh.caudate
37	lh.putamen	78	rh.putamen
38	lh.pallidum	79	rh.pallidum
39	lh.amygdala	80	rh.amygdala
40	lh.hippocampus	81	rh.hippocampus
41	lh.accumbens	82	rh.accumbens
