# Supplementary material for: Predicting Surgery Targets in Temporal Lobe Epilepsy through Structural Connectome Based Simulations
Source: PLoS Comput Biol. 2015 Dec 10;11(12):e1004642. doi: 10.1371/journal.pcbi.1004642 (PMC4675531; doi:10.1371/journal.pcbi.1004642)
Supplement: S2 Table — This table shows a list of the 3 nodes which were consistently the earliest to escape to a seizure state for each individual in patients and controls, ranked in the order of number of appearances. It additionally includes patient age and gender information, where M indicates a male and F female. The numerical code for the node regions is explained in the previous table. Where there is a NaN (‘Not a Number’) label instead of a number there were only one or two nodes which were consistently the earliest to escape. (PDF) [file pcbi.1004642.s004.pdf]

Controls	Age	Gender	Nodes			Patients	Age	Gender	Nodes		
1	34	M	75	35	27	1	44	F	37	10	22
2	48	F	48	45	12	2	43	F	39	80	32
3	35	F	31	32	NaN	3	45	F	21	53	62
4	55	F	39	28	48	4	23	F	39	27	32
5	47	M	9	33	69	5	33	F	39	75	34
6	18	F	62	21	34	6	54	F	80	11	68
7	39	M	69	73	NaN	7	49	M	39	21	47
8	33	F	67	76	75	8	46	M	32	65	39
9	54	F	53	37	78	9	32	M	32	6	15
10	50	F	30	28	65	10	44	M	39	35	80
11	61	F	30	71	78	11	45	F	80	78	76
12	27	M	36	64	26	12	48	F	11	39	52
13	37	M	5	79	64	13	32	F	34	68	55
14	44	M	81	73	23	14	22	M	75	34	23
15	46	M	42	48	80	15	24	F	15	39	63
16	42	F	12	53	28	16	54	F	39	35	76
17	53	M	19	74	27	17	27	M	37	79	75
18	46	F	29	27	68	18	39	M	35	27	39
19	32	M	72	NaN	NaN	19	39	F	28	63	11
20	22	M	34	17	61	20	62	F	45	29	27
21	23	F	81	38	78	21	68	F	39	80	35
22	45	M	4	76	68	22	29	F	12	23	39
23	26	M	20	63	57						
24	67	F	35	34	23						
25	41	M	35	34	76						
26	46	M	66	81	NaN						
27	45	F	34	19	37						
28	31	F	72	23	NaN						
29	24	F	69	27	23						
30	70	F	8	14	76						
31	24	F	28	60	NaN						
32	19	M	22	71	28						
33	36	M	11	18	66						
34	49	M	70	7	75						
35	48	F	68	78	10						
36	21	M	81	58	NaN						
37	58	F	78	54	37						
38	45	F	70	68	64						
39	39	F	63	50	23						
