# Supplementary material for: Predicting Surgery Targets in Temporal Lobe Epilepsy through Structural Connectome Based Simulations
Source: PLoS Comput Biol. 2015 Dec 10;11(12):e1004642. doi: 10.1371/journal.pcbi.1004642 (PMC4675531; doi:10.1371/journal.pcbi.1004642)
Supplement: S3 Table — Fibre length measures and surface areas were checked and documented to look for any evidence of influence. The table shows z scores found from mean fibre lengths normalised by maximum fibre length and z scores from surface area data. These standard scores show the deviation from the main distribution and were found for the mean lengths of fibres connected to the fastest nodes, as well as for the surface areas of the fastest nodes. These measures were checked for all subjects and also within subject groupings of patients and controls. (PDF) [file pcbi.1004642.s005.pdf]

	Z-scores, from mean fibre lengths.			Z-scores, from Surface areas.		
Node	All subjects	Patients only	Controls only	All subjects	Patients only	Controls only
lh.amygdala	0.081293759	0.08728586	0.08728586	-0.381633558	-0.505552189	-0.32925328
lh.temporalpole	-0.43522108	-0.431368087	-0.431368087	-1.00110727	-1.038397738	-1.013346412
rh.amygdala	0.029684499	0.022937121	0.022937121	-0.34154629	-0.36831074	-0.338419951
lh.putamen	0.342168971	0.330502463	0.330502463	0.012017353	-0.003411879	0.020420299
lh.lateralorbitofrontal	0.169811105	0.162182207	0.162182207	0.254764458	0.315429124	0.231267989
rh.thalamus	0.788406376	0.766479009	0.766479009	-0.446216049	-0.480083291	-0.44270331
rh.superiorfrontal	0.296487181	0.275118975	0.275118975	2.706638868	2.71962188	2.785404806
lh.thalamus	0.779128537	0.762401996	0.762401996	-0.491558856	-0.552158355	-0.475577872
rh.insula	-0.162332521	-0.166553994	-0.166553994	0.040592048	0.005854523	0.0599382
lh.insula	-0.128922293	-0.133526555	-0.133526555	0.006081899	-0.012513789	0.015943881
rh.hippocampus	-0.159827477	-0.172577035	-0.172577035	-1.136566035	-1.157983676	-1.161338968
rh.superiortemporal	-0.377072693	-0.373833561	-0.373833561	0.730859341	0.736831107	0.750845745
lh.superiorparietal	0.471064183	0.482228282	0.482228282	1.67738011	1.764661549	1.684990839
rh.inferiorparietal	0.209683711	0.206950634	0.206950634	1.758886907	1.814064084	1.785767197
rh.superiorparietal	0.556655671	0.559292882	0.559292882	1.718027846	1.78333143	1.738351415
