# Supplementary material for: Predicting Surgery Targets in Temporal Lobe Epilepsy through Structural Connectome Based Simulations
Source: PLoS Comput Biol. 2015 Dec 10;11(12):e1004642. doi: 10.1371/journal.pcbi.1004642 (PMC4675531; doi:10.1371/journal.pcbi.1004642)
Supplement: S4 Table — Additional information recorded about the patients included in this study. FS in the Early insult column is short for Febrile Seizures, and the seizure types recorded are simple partial seizures (SPS), complex partial seizures (CPS) and secondary generalised tonic clonic seizures (SGTCS). (PDF) [file pcbi.1004642.s006.pdf]

Patient	Age at onset	Seizure type	Early insult
1	15	SPS, CPS	None
2	1	CPS,SGTCS	None
3	21	CPS	None
4	13	CPS,SGTCS	None
5	13	SPS, CPS, SGTCS	None
6	12	CPS, SGTCS	Lesion
7	15	CPS	FS, Meningitis
8	3	SPS, CPS	FS
9	13	SPS, CPS	FS
10	11	SPS, CPS	None
11	3	CPS	FS
12	30	SPS, CPS, SGTCS	Meningitis
13	29	SPS	None
14	1	SPS, CPC	None
15	1	CPS	FS
16	14	SPS,CPS,SGTCS	Encephalitis
17	18	SPS,SGTCS	FS
18	17	CPS,SGTCS	Encephalitis
19	36	CPS	None
20	21	SPS, CPS	None
21	47	SPS, SGTCS	None
22	15	CPS	FS
